# Preoperative Comorbidity Correlates Inversely with Survival after Intestinal and Multivisceral Transplantation in Adults

**DOI:** 10.1155/2013/202410

**Published:** 2013-04-15

**Authors:** Rajesh Sivaprakasam, Takahashi Hidenori, Charlotte Pither, Seigo Nishida, Andrew J. Butler, Eddie R. Island, Jung Moon, Muhammad Dawwas, Simon M. Gabe, Neville V. Jamieson, Andreas G. Tzakis, Stephen J. Middleton

**Affiliations:** ^1^Department of Gastroenterology and Transplantation, Addenbrooke's, Cambridge University Teaching Hospital, Cambridge CB2 0QQ, UK; ^2^Transplantation Surgery, Addenbrooke's, Cambridge University Teaching Hospital, Cambridge CB2 0QQ, UK; ^3^Division of Transplantation, Department of Surgery, University of Miami School of Medicine, Miami, FL 33136, USA; ^4^The Lennard-Jones Intestinal Failure Unit, St. Mark's Hospital and Academic Unit, Harrow HA1 3UJ, UK; ^5^Transplant Center, Cleveland Clinic Florida, 2950 Cleveland Clinic Boulevard, Weston, FL 33331, USA

## Abstract

We investigated the relationship between preoperative comorbidity and postoperative survival after intestinal transplantation. Each patient received a score for preoperative comorbidity. Each comorbidity was given a score based on the degree it impaired function (score range 0–3). A total score was derived from the summation of individual comorbidity scores. Patients (72 adults (M : F, 33 : 39)) received an isolated intestinal graft (27) or a cluster graft (45). Mean (standard deviation) survival was 1501 (1444) days. The Kaplan-Meier analysis revealed a significant inverse association between survival and comorbidity score (logrank test for trend, *P* < 0.0001). Patients grouped into comorbidity scores of 0 and 1, 2 and 3, 4 and 5, 6, and above had hazard ratios (95% confidence intervals) for death (compared to group 0 + 1), which increased with comorbidity scores: 1.945 (0.7622–5.816), 5.075 (3.314–36.17), and 13.77 (463.3–120100), respectively, (*P* < 0.0001). Receiver-operator curves at 1, 3, 5, and 10 years postoperative had “C” statistics of 0.88, 0.85, 0.88, and 0.92, respectively. When evaluating patients for transplantation, the degree of comorbidity should be considered as a major factor influencing postoperative survival.

## 1. Introduction

Over the last decade, intestinal transplantation has become an established treatment modality in the management of intestinal failure. The preoperative status of patients has been found to influence the outcome of surgery [1], and scores developed to semiquantify this [2, 3] have been used in routine practice to facilitate preoperative risk assessment. There has been considerable improvement in postoperative survival over the last 20 years. Much of this improvement has arisen from advances in immunosuppressive regimes and better preoperative preparation and postoperative care of patients [4]. Although survival after transplantation remains inferior to that on parenteral nutrition (PN), the gap is closing, and as a consequence patients are being considered for transplantation at an earlier stage. Potential improvement in quality of life is also now being factored in to the decision regarding a patient's suitability for transplantation. It has therefore become very important to accurately assess patient's individual survival chance. In our routine clinical practice, we have considered preoperative comorbidity to have a negative influence on postoperative survival [3]. We have semiquantified comorbidity for each patient and compared this to postoperative survival to determine the relationship.

## 2. Methods

Preoperative comorbidity was transformed into a numerical score to allow service analysis to take place. This work was conducted according to the requirements of the institution. The score was compared to subsequent survival. Comorbidity factors included were loss of venous access and impairment of organs or systems not corrected by transplantation. Each factor was scored 0–3. A score of 3 indicated a functional abnormality not fully corrected by treatment of a severity approaching a contraindication for transplantation. A score of 1 describes a characteristic which does not present an immediate risk but which may develop into a risk factor in the future. A score of 2 indicates an abnormality of function which is corrected by ongoing treatment and conveys moderate risk (Table 1).

Following the loss of 2 large venous access sites suitable for parenteral nutrition and high flow infusions (i.e., internal jugular, subclavian, and femoral vein), each subsequent loss was given a score of 1.

## 3. Surgery

Patients were selected for transplantation according to the conventional indications as previously published [5, 6]. In summary, these were irreversible intestinal failure and inability to continue parenteral nutrition, due to such conditions as loss of venous access and parenteral nutrition-induced liver disease. Other indications included the need for extensive evisceration to remove desmoid tumors. Patients received an intestinal graft either in isolation or as part of a cluster of abdominal organs including liver (multivisceral) or excluding liver (modified multivisceral transplant). All patients received induction therapy with either Alemtuzumab (Campath 1H), Daclizumab (Zenapax), or a few with glucocorticosteroids. The surgical techniques and postoperative care were in keeping with those previously described [5].

## 4. Data Collection

The data were collected prospectively during routine preoperative evaluation at the transplant centres at the University of Cambridge, United Kingdom, and University of Miami, United States of America, from 1997 to 2003. The analysis was undertaken retrospectively. Patient data were consecutively entered into a database as a routine at each institution from which data were acquired for the analysis. Where comorbidity data were incomplete, patients were excluded from the analysis; this was undertaken without knowledge of their subsequent survival.

## 5. Statistics

The primary outcome measured was median recipient survival with 95% confidence interval (CI) and was calculated by Kaplan-Meier (KM) approach with comparison using logrank test. The accuracy of the comorbidity score to predict postoperative survival was tested by using Harrell's C statistic which estimates the proportion of accurate predictions. A Harrell's C index varies from 0.5 to 1 reflecting no discrimination to perfect discrimination, respectively.

Student's *t*-test for analysis of variance was used.

## 6. Results

The donor and patient demographics are detailed in (Tables 2 and 3, resp.). A total of 72 adults (M : F, 33 : 39) were included in the study.

Patients received either an isolated intestinal graft (*n* = 27) or intestine in combination with other organs including liver (multivisceral) (*n* = 27) or excluding liver (modified multivisceral) (*n* = 18). In 31 patients, the large intestine was also transplanted, and 6 received a kidney. Mean (SD) survival of the group was 1501 (1444) days. No repeat transplantations were included in the cohort.

The cause of intestinal failure in recipients was most commonly short gut syndrome (33%), and the most frequent procedure in this cohort was multivisceral transplantation (intestine, liver, and additional organs) in 38%, followed by isolated intestine (37%), and modified multivisceral (intestine and other organs excluding liver) 25% (Table 3). 

Most patients received Alemtuzumab (Campath 1H) induction immunosuppression, and the mean (SD) number of rejection episodes per patient was 1.22 ± 1.34 (Table 4).

### 6.1. Kaplan-Meier Survival Analysis

**Figure 1:**
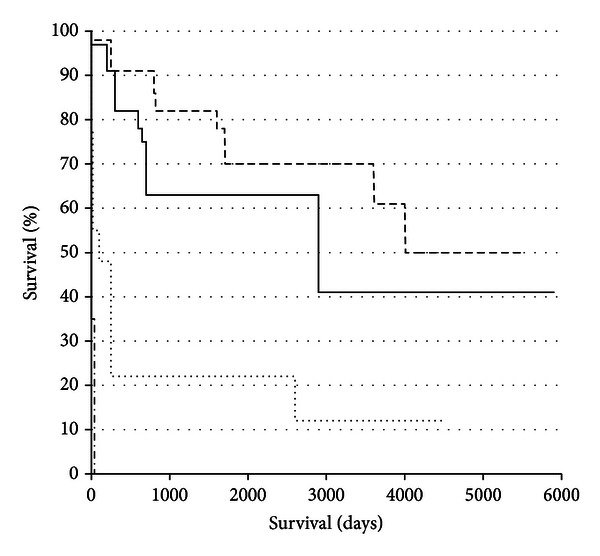
Km survival curves were plotted for patients grouped according to comorbidity score (dashed line: comorbidity 0 + 1; continuous line: comorbidity 2 + 3; dotted line: comorbidity 4 + 5; and dashed/dotted line: comorbidity >6). Logrank test for trend indicated a significant increase in rate of death with comorbidity score, and compared with comorbidity 0 + 1, the hazard ratios for death were found to increase with comorbidity score, 1.94 (0.76–5.82), 5.07 (3.31–36.17), and 13.77 (46.3–120100), respectively; the difference became significantly greater than group 0 + 1 at group 4 + 5 (*P* < 0.0001).

Individual patient comorbidity scores and their postoperative survivals were found to correlate inversely (*r*_*s*_  −0.72; *P* < 0.0001). The Kaplan-Meier (KM) curve analysis of the cumulative recipient survival revealed a significant inverse association between patient survival and comorbidity score as evaluated by the logrank test for trend (*P* < 0.0001) (Figure 1). For the purposes of comparing survival chance, patients were grouped into comorbidity scores of 0 and 1, 2 and 3, 4 and 5, 6, and above. The hazard ratio (95% CI) for death (compared to group 0 + 1) was found to increase as the comorbidity score increased: 1.945 (0.7622–5.816), 5.075 (3.314–36.17), and 13.77 (463.3-120100), respectively; this became significantly greater than group 0 + 1 at group 4 + 5 (*P* < 0.0001) (Figure 1).

The survival of patients receiving isolated intestinal grafts was compared to those who underwent multivisceral transplantation. Kaplan-Meier analysis revealed no difference in survival between patients receiving isolated and multivisceral transplantation: logrank (Mantel-Cox) test: *P* = 0.43; hazard ratio: 0.8129 (0.4599 to 1.366). Median survival for isolated transplants (2007 days) was similar to that for multivisceral grafts (3107 days).

### 6.2. Receiver-Operator Curve Analysis

**Figure 2:**
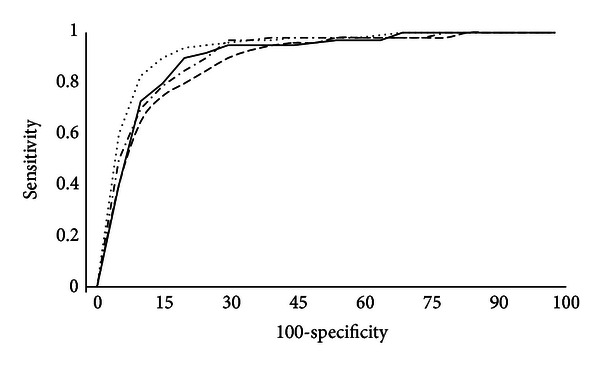
Receiver-operator curves were calculated to assess accuracy of survival prediction at 1, 3, 5, and 10 years postoperative (continuous line, dashed line, dashed/dotted line, and dotted line, resp.). The accuracy of the comorbidity score is indicated by the area under the curve (Harrell's “C” statistic) and was very good or excellent for predictions at each time frame with associated “C” statistics of 0.88, 0.85, 0.88, and 0.92, respectively.

The accuracy of the score to predict postoperative survival was evaluated using the receiver-operator characteristic analysis. A receiver-operator curve (ROC curve) was constructed for survival prediction at 1, 3, 5, and 10 years. The Harrell's C statistic was calculated for each curve and represents the overall accuracy when assessed in terms of specificity and sensitivity at all possible points. The C statistic indicated very good or excellent accuracy of the comorbidity score at all times frames (Figure 2, Table 5).

## 7. Discussion

We recognise that the interpretation of these data should be undertaken in the context that there will be differences in the protocols of transplant centres which might have an influence on the relative importance of comorbidity on survival. For instance, the majority of patients in our cohort received Alemtuzumab as induction therapy which may have made the impact of comorbidity more significant. There will also be additional influences on survival, particularly in the long term, that are not included in this analysis. Nevertheless, in this cohort of patients, our results indicate that the degree of preoperative comorbidity appears to predict posttransplant survival following small intestinal and multivisceral transplantation in adults. The predictive value of the comorbidity score might allow better patient selection, focus on pretransplant optimization, and facilitate informed consent for the procedure. Patients who have a low comorbidity score might be considered for earlier transplantation or even preemptive transplantation, where transplantation is offered at the time of developing intestinal failure. This would be particularly appropriate for those with high risk circumstances, where they are likely to have a reduced life expectancy on PN [6], such as pseudoobstruction and systemic sclerosis and possibly ultrashort bowel syndrome (<30 cm of jejunum), rather than waiting for them to develop the traditional indications through complications of their PN. In better performing centres, it might be reasonable to offer patients preemptive transplantation on the basis of improving a very poor quality of life (QOL). Patients in comorbidity groups 0 + 1 have a 10-year survival similar or better than unselected patients on PN. However, before this becomes common practice, it will be important to develop methods of determining which patients will benefit in terms of QOL from transplantation, as it is clear that certain aspects of patient's lives do not improve with transplantation [7]. Furthermore, the timing of transplantation might be influenced by the desire to limit comorbidity to keep the comorbidity score low. For instance, a patient with early comorbidity in several body systems might benefit from early transplantation before these deteriorate; conversely, patients with active comorbidity might have a better survival chance if transplantation is delayed until they receive treatment to stabilise concurrent illness.

As postoperative survival improves poor quality of life, it is likely to become a more frequent indication for transplantation. Under these circumstances, it will be crucial to predict postoperative survival for individual patients to better evaluate the risk/benefit ratio of transplantation and facilitate informed consent, and the comorbidity score might also be of considerable use for this purpose. As the role of other factors, such as the presence of HLA antibodies, becomes clearer, this scoring system may be improved by their inclusion [8].

In conclusion, the numeric score (comorbidity score) developed to allow us to semiquantify preoperative comorbidity appears to be an accurate and convenient method of assessing risk using preoperative risk factors. A future multicentre international prospective study to evaluate this survival risk factor is important to confirm our initial observation and may lead to a useful clinical tool in the selection and timing of patients for transplantation.

## Figures and Tables

**Table 1:** Pretransplant risk assessment score for intestinal and multivisceral transplantation in adults. The score was assigned to the severity of each comorbidity resulting in a cumulative comorbidity score.

Score	Degree of comorbidity
0	Normal

1	Comorbidity with no significant functional impairment requiring treatment: (mild hypertension, slight glucose intolerance—diet controlled, mild exercise-induced asthma, renal impairment—slightly impaired GFR reduced by 10% only, smoker without respiratory dysfunction, and past history of depressive illness)

2	Comorbidity requiring treatment to prevent functional impairment—deemed to present a slightly increased surgical risk: (treated hypertension, mild coronary artery disease without symptoms, DM-oral hypoglycemics, mild asthma—stable on intermittent therapy, renal impairment—moderately reduced GFR 10–25%, smoker with mild respiratory dysfunction but no symptomatic limitation, treated and resolved depression).

3	Comorbidity that despite treatment causes functional impairment and presents a moderate risk to surgery: (poorly controlled hypertension, angina controlled by Tx—minor vessel disease, DM—insulin dependent, renal impairment—moderately reduced GFR 25–50%, treated but unresolved depression, COPD with mild symptoms requiring occasional treatment during exacerbations)

Venous access: after loss of two major venous access points each additional loss scores one point.

**Table 2:** Donor demographics.

Variables	Results
Mean age (yrs ± SD)	21.18 ± 11.27
Mean weight (kgs ± SD)	57.37 ± 18.61
CMV mismatch (%)	21.16%
Cold ischemic time (min)	434 ± 117.1

**Table 3:** Recipient demographics.

Variables	Results
Mean age (yrs ± SD)	35.76 ± 10.63
Sex (M : F)	1 : 1.49
Race (%)	
Caucasian	75.06%
Afro-Caribbean	14.42%
Hispanic	10.52%
Mean weight (kgs ± SD)	51.95 ± 13.92
Mean time on the waiting list (days ± SD)	68.95 ± 18.47
Pre-op place (hospital : home)	1 : 1.35
Pre-Tx albumin (g/dL ± SD)	3.268 ± 0.688
Thrombosed veins (mean ± SD)	1.495 ± 1.291
HLA mismatch (mean ± SD)	1.035 ± 1.076
Indication for transplant (%)	
Short gut	33.33%
Motility disorders	16.67%
Neoplasms	18.05%
Mucosal defects	13.51%
Others	18.33%
Types of transplant (%)	
Multivisceral (MVT)	38.37%
Modified MVT	24.65%
Intestine alone	36.98%
Warm ischemic time (minutes ± SD)	39.35 ± 12.07

**Table 4:** Peri- and postoperative data.

Variables	Results
Induction agents (%)	
Corticosteroids	13.90%
Daclizumab (Zenapax)	16.66%
Alemtuzumab (Campath)	69.44%
Number of rejection episodes (mean ± SD)	1.22 ± 1.34
Severity of rejection episodes per patient (mean ± SD)	
Mild	1.419 ± 0.662
Moderate	0.391 ± 0.718
Severe	0.318 ± 0.547
Rejection-free period (days) (mean ± SD)	357.63 ± 21.54
Posttransplant hospital stay (days) (mean ± SD)	60.69 ± 45.57

**Table 5:** 

Receiver-operator curve (ROC) analysis for predicting death at postoperative time intervals.						
Time point assessed	Area under ROC curve (Harrel's “C” statistic)	Standard error	95% confidence interval	Significance (*P* value)	Number of control subjects	Number of patients
1 yr	0.88	0.049	0.788–0.981	<0.0001	34	20
3 yrs	0.85	0.054	0.742–0.954	<0.0001	28	28
5 yrs	0.88	0.057	0.7761–0.9905	<0.0001	13	29
10 yrs	0.92	0.040	0.8454–1.003	<0.0001	10`	33

## Abbreviations

SD: Standard deviation
CI: Confidence interval
Pn: Parenteral nutrition
DM: Diabetes mellitus
COPD: Chronic obstructive pulmonary disease
KM: Kaplan-Meier
M: Male
F: Female
*r*_*s*_: Spearman's correlation coefficient
ROC: Receiver-operator characteristic
QOL: Quality of life
Yrs: Years
g: Grams
dL: Decilitre
MVT: Multivisceral.
